# Development and validation of a model for predicting the early occurrence of RF in ICU-admitted AECOPD patients: a retrospective analysis based on the MIMIC-IV database

**DOI:** 10.1186/s12890-024-03099-2

**Published:** 2024-06-26

**Authors:** Shiyu Hu, Ye Zhang, Zhifang Cui, Xiaoli Tan, Wenyu Chen

**Affiliations:** 1 Jiaxing University Master Degree Cultivation Base, Zhejiang Chinese Medical University, Jiaxing, China; 2 Department of Respiratory medicine, Affiliated Hospital of Jiaxing University, Jiaxing, China; 3 Department of General Medicine, Jiaxing, China; 4 Department of Respiratory medicine, Dongzhimen Hospital, Beijing University of Chinese Medicine, Jiaxing, China

**Keywords:** Acute exacerbation of chronic obstructive pulmonary disease, Respiratory failure, Nomogram, MIMIC-IV, ICU

## Abstract

**Background:**

This study aims to construct a model predicting the probability of RF in AECOPD patients upon hospital admission.

**Methods:**

This study retrospectively extracted data from MIMIC-IV database, ultimately including 3776 AECOPD patients. The patients were randomly divided into a training set (*n* = 2643) and a validation set (*n* = 1133) in a 7:3 ratio. First, LASSO regression analysis was used to optimize variable selection by running a tenfold k-cyclic coordinate descent. Subsequently, a multifactorial Cox regression analysis was employed to establish a predictive model. Thirdly, the model was validated using ROC curves, Harrell’s C-index, calibration plots, DCA, and K-M curve.

**Result:**

Eight predictive indicators were selected, including blood urea nitrogen, prothrombin time, white blood cell count, heart rate, the presence of comorbid interstitial lung disease, heart failure, and the use of antibiotics and bronchodilators. The model constructed with these 8 predictors demonstrated good predictive capabilities, with ROC curve areas under the curve (AUC) of 0.858 (0.836–0.881), 0.773 (0.746–0.799), 0.736 (0.701–0.771) within 3, 7, and 14 days in the training set, respectively and the C-index was 0.743 (0.723–0.763). Additionally, calibration plots indicated strong consistency between predicted and observed values. DCA analysis demonstrated favorable clinical utility. The K-M curve indicated the model’s good reliability, revealed a significantly higher RF occurrence probability in the high-risk group than that in the low-risk group (*P* < 0.0001).

**Conclusion:**

The nomogram can provide valuable guidance for clinical practitioners to early predict the probability of RF occurrence in AECOPD patients, take relevant measures, prevent RF, and improve patient outcomes.

**Supplementary Information:**

The online version contains supplementary material available at 10.1186/s12890-024-03099-2.

## Introduction

Chronic obstructive pulmonary disease (COPD), a significant group of chronic respiratory diseases, has the characteristics of persistent as well as often progressive airflow obstruction because of abnormalities in the airways and/or alveoli, resulting in chronic respiratory symptoms [1]. This disease has a high prevalence, disability rate, and mortality rate. Research statistics indicated that 212.3 million of the global population were affected by COPD in 2019, with 74.4 million experiencing disability due to COPD, and 3.3 million deaths attributed to the disease [2].

Acute exacerbation of chronic obstructive pulmonary disease (AECOPD), a frequent clinical event in the natural course of COPD, is closely associated with a decline in the patient’s health status, reduced quality of life, and an elevated risk of mortality [3]. AECOPD is primarily distinguished by exacerbated symptoms of dyspnea, increased coughing and sputum production, possibly accompanied by rapid breathing and increased heart rate. In cases of further deterioration, patients may experience cardiopulmonary dysfunction and metabolic disturbances, ultimately leading to respiratory failure (RF) [1]. The occurrence of RF is the most common risk factor for repeated hospitalizations, adverse outcomes, and mortality in COPD patients [4]. Early assessment of the probability of RF in AECOPD patients may aid in preventing the occurrence of RF and promoting early treatment, potentially reducing the risk of disease progression, and promoting the prognosis, as well as improving quality of life in people with COPD. However, there is currently a lack of effective tools for predicting the probability of RF in AECOPD patients.

## Materials and methods

### Data source and ethics statement

Our study is a retrospective analysis, and all data were sourced from the Intensive Care Medical Information Mart for Intensive Care IV 2.2 (MIMIC-IV version 2.2) database (https://physionet.org/content/mimiciv/2.2/). This database is a publicly available clinical intensive care database, collaboratively developed by the Massachusetts Institute of Technology’s Laboratory for Computational Physiology, Beth Israel Deaconess Medical Center (BIDMC), and Philips. It comprises a large open-access, multi-parameter structured critical care data, including demographic information, vital signs, laboratory indicators, and medication usage. We have completed the necessary coursework and personal training examinations to access and use this database, obtained the corresponding certificates (certificate number: 52,663,507), and have been granted permission for database access. All the protected health information of patients in the MIMIC project has been anonymized, and the research does not involve ethical concerns.

### Study population

**Fig. 1:**
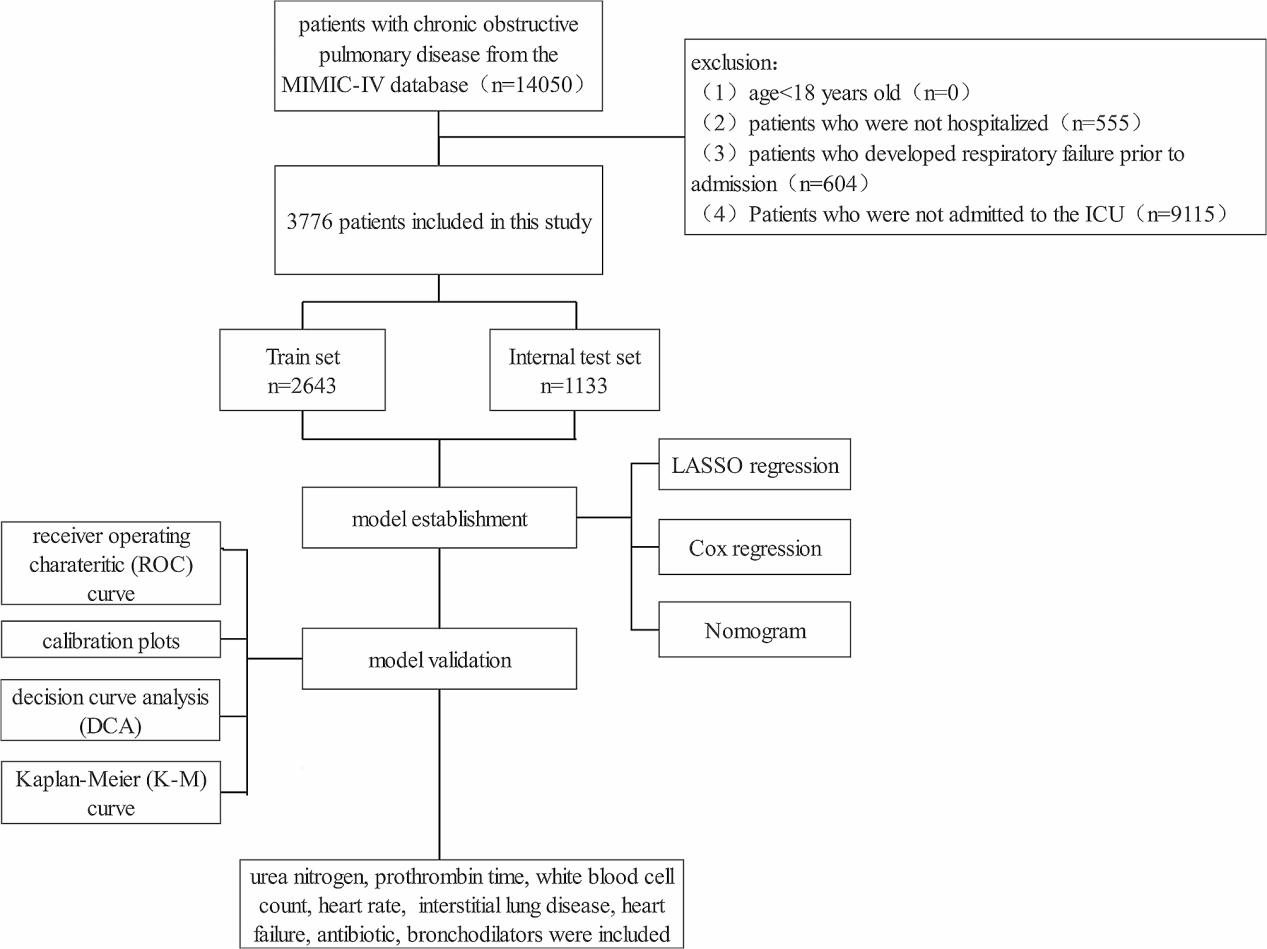
Inclusion and exclusion flowchart of the study

Data from MIMIC-IV were extracted, identifying 14,050 individuals diagnosed with COPD using International Classification of Diseases, Ninth Revision (ICD-9) codes (49,120, 49,121, 49,122, 496) and International Classification of Diseases, Tenth Revision (ICD-10) codes (J44, J440, J441, J449). The study excluded individuals as follows: (1) patients younger than 18 years old, (2) patients without hospitalization records, (3) individuals who experienced RF before admission, and (4) patients not admitted to the Intensive Care Unit (ICU). The final analysis included 3,776 patients. Additionally, for patients with multiple admissions, we only extracted the data from their first hospitalization. Figure 1 illustrates a detailed overview of the patient selection process.

### Data extraction

The Navicat Premium software (version 16.0, https://navicat.com.cn) was used to extract clinical data of the study population from MIMIC-IV. Detailed patient information like age, BMI, and gender, was collected. Additionally, we gathered the first measured blood test results after patient admission, including (i) complete blood count: platelet count (PLT), red blood cell count (RBC), white blood cell count (WBC), neutrophil count (Ncell), and lymphocyte count (Lcell); (ii) biochemical indexes: serum creatinine (Scr), sodium concentration (Na+), potassium concentration (K+), glucose, blood urea nitrogen (BUN), triglyceride, alanine aminotransferase (ALT), aminotransferase (AST), albumin, total bilirubin (TBil), indirect bilirubin (IBil), hemoglobin A1c (HbA1c), total cholesterol (TC), low-density lipoprotein (LDL), and high-density lipoprotein (HDL); (iii) coagulation parameters: activated partial thromboplastin time (APTT) and prothrombin time (PT), D-Dimer. Vital signs: heart rate (HR) and respiratory rate (RR). Comorbidities such as asthma, interstitial lung disease (ILD), heart failure (HF), diabetes mellitus (DM), and cerebrovascular disease (CVD), Liver cirrhosis, Viral hepatitis. Medication usage, including the use of glucocorticoids, antibiotics, bronchodilators, and albumin, was recorded. All arterial blood gas analysis results during hospitalization, such as partial pressure of oxygen (PO2) and partial pressure of carbon dioxide (PCO2), were also collected. Furthermore, to prevent reverse causation, data after the occurrence of RF were considered invalid. The results of forty metrics were collected. The data used in this study were derived from inpatient records, thus there was no loss to follow-up.

### Statistical analysis

For continuous variables conforming to a normal distribution, the mean and standard deviation (SD) were calculated, and for those not following a normal distribution, the median and interquartile range (IQR) were calculated. Counts as well as percentages (%) were employed to express categorical variables. We employed student’s t-tests or non-parametric tests to compare continuous variables based on their distribution. The results of the normality test are exhibited in Supplementary Table 1. Pearson’s chi-square test or Fisher’s exact test was adopted to compare categorical variables. Multiple imputation was applied to handle missing data when the missing values were less than 20%, and data with missing values exceeding 20% were excluded. Finally, 26 variables were identified. A detailed overview of the missing data for all variables is provided in Supplementary Table S2.

All statistical analyses were conducted by using the R software (http://www.R-project.org; version 4.2.3). All the tests in the present research were two-sided, and a *P*-value of < 0.05 indicated statistical significance. The ‘missForest’ package was used for multiple imputations of missing values, the ‘caret’ package was employed to split the data into a training or validation set, and the ‘tableone’ package was utilized to analyze the baseline characteristics of patients in both sets and conduct intergroup comparisons. We performed the least absolute shrinkage and selection operator (LASSO) regression analysis with the ‘glmnet’ package and multifactorial Cox regression analysis with the ‘glm’ package. The ‘pROC’ package, ‘ggROC’ package, and ‘fbroc’ package were used for drawing the receiver operating characteristic (ROC) curves and calculating areas under the curve (AUC). The ‘rms’ package, specifically the ‘val.prob’ function and ‘calibrate,’ was used for generating calibration curves and the nomogram. The ‘rmda’ package was employed for drawing decision curve analysis (DCA), and the ‘survminer’ package was used to create Kaplan-Meier(K-M) curves.

### Nomogram construction and validation

According to the inclusion criteria, our study ultimately included 3,776 patients. Initially, they were randomly divided into a training set (*n* = 2,643) or a validation set (*n* = 1,133) at a ratio of 7:3. The selection of predictive variables involved two steps. In the first step, LASSO regression analysis [5] was adopted to explore potential confounding factors associated with the probability of the occurrence of RF in individuals with AECOPD. LASSO regression, proposed by Robert Tibshirani [6], refines the model by constructing a penalty function, resulting in a more concise model that compresses some coefficients and sets some coefficients to zero, thus retaining the advantages of subset contraction. In the second step, a nomogram model was established using the variables selected by LASSO regression, and a multifactorial Cox regression analysis was performed.

The validation of the predictive model primarily involved four processes: discrimination evaluation, calibration evaluation, clinical applicability evaluation and rationality analysis. In our study, the discrimination of the model was assessed using the concordance index (C-index) as well as AUC. The calibration plot was employed to evaluate the model’s calibration, and DCA was employed to assess the clinical utility of the model. Additionally, we used K-M curves for model performance verification.

### Study outcomes

The primary outcomes of this study are the probabilities of RF occurring in AECOPD patients within 3, 7, and 14 days after hospital admission. The measurement time for PO_2_ and PCO_2_ was carefully documented. RF was defined as PO_2_ < 60 mmHg, with or without PCO_2_ ≥ 50 mmHg.

## Results

### Characteristics of the included patients

From the MIMIC-IV 2.2 database, we retrospectively identified 14,050 patients diagnosed with COPD, ultimately incorporating 3,776 eligible patients in the current analysis. Table 1 presents the baseline characteristics of all the included patients. The patients included in our research had a median age of 71 years [62.0; 79.0], including 2,072 males (54.9%), with 1,045 (27.7%) experiencing RF. The training set and validation set exhibited good comparability among patients.

**Table 1:** Baseline characteristics of the study population

Variables	Total (*n* = 3776)	validation set (*n* = 1133)	training set *N* = 2643	*P*-value	Test
**Age, years, median [IQR]**	71.0 [62.0;79.0]	71.0 [62.0;79.0]	71.0 [63.0;79.0]	0.234	nonnorm
**Gender**				0.346	
Female	1704 (45.1%)	525 (46.3%)	1179 (44.6%)		
Male	2072 (54.9%)	608 (53.7%)	1464 (55.4%)		
**Laboratory test, median (IQR)**					
BUN, mg/dL	20.0 [14.0;30.0]	20.0 [14.0;31.0]	20.0 [14.0;30.0]	0.869	nonnorm
Scr, mg/dL	0.98 [0.70;1.30]	0.90 [0.70;1.30]	1.00 [0.70;1.30]	0.468	nonnorm
Glucose, mg/dl	124 [101;159]	124 [102;157]	123 [101;160]	0.842	nonnorm
HbA1c, g/dL	11.3 [9.80;12.7]	11.3 [9.70;12.6]	11.3 [9.80;12.8]	0.416	nonnorm
Na^+^, mEq/L	139 [136;141]	139 [136;141]	139 [136;141]	0.824	nonnorm
K^+^, mEq/L	4.11 [3.80;4.50]	4.15 [3.80;4.60]	4.10 [3.80;4.50]	0.683	nonnorm
APTT, sec	31.3 [27.6;38.7]	31.0 [27.5;37.9]	31.4 [27.7;39.2]	0.173	nonnorm
PT, sec	13.6 [12.1;16.0]	13.6 [12.1;15.8]	13.6 [12.1;16.2]	0.492	nonnorm
WBC, K/µL	10.2 [7.60;13.7]	10.5 [7.80;13.9]	10.1 [7.50;13.6]	0.137	nonnorm
PLT, K/µL	206 [155;264]	211 [157;271]	205 [154;260]	0.066	nonnorm
RBC, K/Μl	3.79 [3.27;4.24]	3.78 [3.24;4.22]	3.79 [3.28;4.25]	0.321	nonnorm
**Comorbidities**					
Asthma, n (%)	260 (6.89%)	78 (6.88%)	182 (6.89%)	1.000	
CVD, n (%)	615 (16.3%)	186 (16.4%)	429 (16.2%)	0.926	
DM, n (%)	1179 (31.2%)	355 (31.3%)	824 (31.2%)	0.955	
HF, n (%)	1346 (35.6%)	392 (34.6%)	954 (36.1%)	0.399	
ILD, n (%)	42 (1.11%)	14 (1.24%)	28 (1.06%)	0.761	
Liver cirrhosis, n (%)	166 (4.40%)	43 (3.80%)	123 (4.65%)	0.275	
Viral hepatitis, n (%)	95 (2.52%)	26 (2.29%)	69 (2.61%)	0.649	
**Medicine**					
Glucocorticoid, n(%)	1211 (32.1%)	349 (30.8%)	862 (32.6%)	0.292	
Bronchodilators, n(%)	1637 (43.4%)	476 (42.0%)	1161 (43.9%)	0.293	
Antibiotic, n(%)	2155 (57.1%)	632 (55.8%)	1523 (57.6%)	0.311	
Usealbumin, n (%)	728 (19.3%)	219 (19.3%)	509 (19.3%)	0.996	
**Vital signs**					
HR, bpm	83.0 [72.0;95.0]	84.0 [72.0;95.0]	83.0 [72.0;95.0]	0.342	nonnorm
RR, insp/min	19.0 [16.0;23.0]	19.0 [16.0;23.0]	19.0 [16.0;23.0]	0.932	nonnorm
**Events**					
RF status, n(%)	1045 (27.7%)	304 (26.8%)	741 (28.0%)	0.472	

**Abbreviations**

HR, Heart rate; RR, Respiratory rate; CVD, Cerebrovascular disease; HF, Heart failure; DM, Diabetes mellitus; ILD, Interstitial Lung Disease; WBC, White blood cell; RBC, Red blood cell; PLT; Platelet; HbA1c, Glycosylated hemoglobin; Scr, Serum creatinine; K^+^, Potassium; Na+, Sodium; BUN, Blood urea nitrogen; APTT, Activated partial thromboplastin time; PT, Prothrombin time; RF, Respiratory failure

### Variable filtering of the training set

We removed data with missing values exceeding 20%. Then, 8 potential predictor variables out of the 26 extracted feature variables were chosen according to the full dataset, which had nonzero coefficients in the LASSO regression (family= “cox”) model. When selecting features to build prediction models, the largest λ at which the mean square error (MSE) falls within one standard error of the minimal MSE was taken into consideration. This process identified potential predictive factors, including BUN, PT, WBC, HR, the presence of ILD, HF, and the use of antibiotics and bronchodilators.

### Construction of the predictive model

**Fig. 2:**
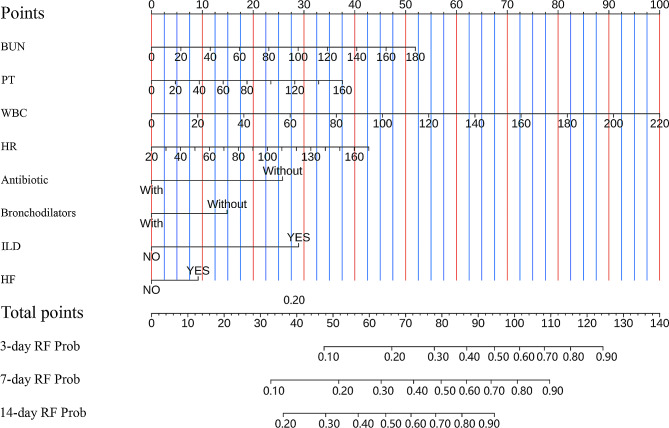
Developed risk nomograms for the probability of RF occurrence in AECOPD patients. RF, Respiratory failure; BUN, Blood urea nitrogen; PT, Prothrombin time; WBC, White blood cell; HR, Heart rate; ILD, Interstitial Lung Disease; HF, Heart failure; Prob, probability

The LASSO regression model verified the eight predictors were the optimal set. These factors were then utilized to construct the Cox regression model. Table 2 displays the findings from the univariate as well as the multivariate analyses. To visualize the model, a nomogram was developed (Fig. 2). The score for each variable corresponds to the score (points) on the upper scoring axis. The total score corresponds to the risk probability of RF in AECOPD patients on the lower axis.

**Table 2:** Univariate and multivariate analyses of Cox-proportional hazards model for the risk of RF

Characteristics	Univariate (HR^1^)	Univariate (95% CI)	*P*-value	Multivariate (HR^1^)	Multivariate (95% CI)	*P*-value
RR	1.019	1.006–1.032	0.005			
HR^2^	1.008	1.004–1.012	0	1.011	1.007–1.015	0
Antibiotics	2.714	2.326–3.166	0	2.636	2.248–3.09	0
GC	1.475	1.253–1.738	0			
Albumin	1.475	1.224–1.776	0			
Bronchodilators	1.92	1.651–2.233	0	1.727	1.474–2.024	0
Viral hepatitis	0.754	0.452–1.257	0.279			
Asthma	0.676	0.483–0.944	0.022			
Liver cirrhosis	1.213	0.894–1.645	0.215			
ILD	2.473	1.506–4.061	0	2.82	1.714–4.641	0
HF	1.481	1.282–1.712	0	1.413	1.216–1.643	0
DM	1.092	0.937–1.272	0.26			
CVD	0.739	0.598–0.914	0.005			
WBC	1.013	1.008–1.018	0	1.017	1.011–1.023	0
Scr	1.103	1.055–1.154	0			
RBC	0.935	0.846–1.034	0.191			
PT	1.016	1.009–1.022	0	1.008	1.001–1.015	0.033
PLT	0.999	0.999-1	0.079			
Na^+^	0.992	0.976–1.007	0.277			
K^+^	1.151	1.038–1.276	0.007			
HbA1c	0.952	0.92–0.984	0.004			
Glucose	1.002	1.001–1.003	0			
BUN	1.012	1.009–1.015	0	1.011	1.008–1.014	0
APTT	1.003	1-1.006	0.033			
Gender (male)	1.021	0.883–1.181	0.775			
Age	1.002	0.996–1.009	0.511			

**Abbreviations**

HR^1^, Hazard Ratio; HR^2^, Heart rate; RR, Respiratory rate; CVD, Cerebrovascular disease; HF, Heart failure; DM, Diabetes mellitus; ILD, Interstitial Lung Disease; WBC, White blood cell; RBC, Red blood cell; PLT; Platelet; HbA1c, Glycosylated hemoglobin; Scr, Serum creatinine; K^+^, Potassium; Na+, Sodium; BUN, Blood urea nitrogen; APTT, Activated partial thromboplastin time; PT, Prothrombin time; RF, Respiratory failure; GC, glucocorticoids

### Validation of predictive models

**Fig. 3:**
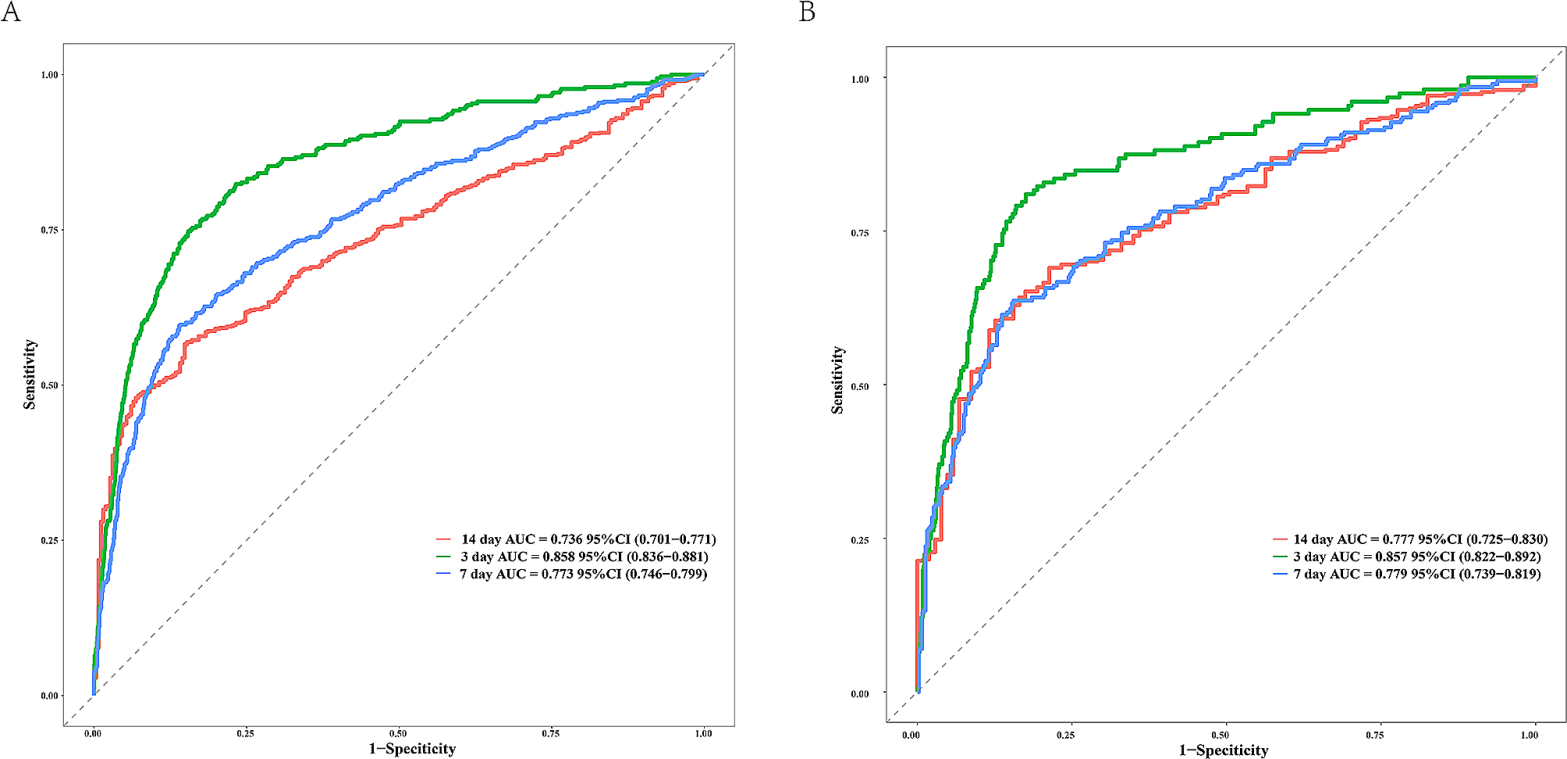
The receiver operating characteristic curve for the train and validation cohorts. (**A**) The ROC curve for the train cohort. (**B**) The ROC curve for the validation cohort. ROC curve, receiver operating characteristic curve

After bias correction, the C-index (95% CI) for the training set and validation set was 0.743 (0.723–0.763) and 0.742 (0.711–0.773), respectively. ROC curve analysis (Fig. 3A and B) revealed that the nomogram had AUC (95% CI) values of 0.858 (0.836–0.881), 0.773 (0.746–0.799), and 0.736 (0.701–0.771) for 3, 7, and 14 days, respectively, in the training set, and 0.857 (0.822–0.892), 0.779 (0.739–0.819), and 0.777 (0.725–0.830), respectively, in the validation set. Both the C-index and AUC indicated that the model had a good discriminative ability.

**Fig. 4:**
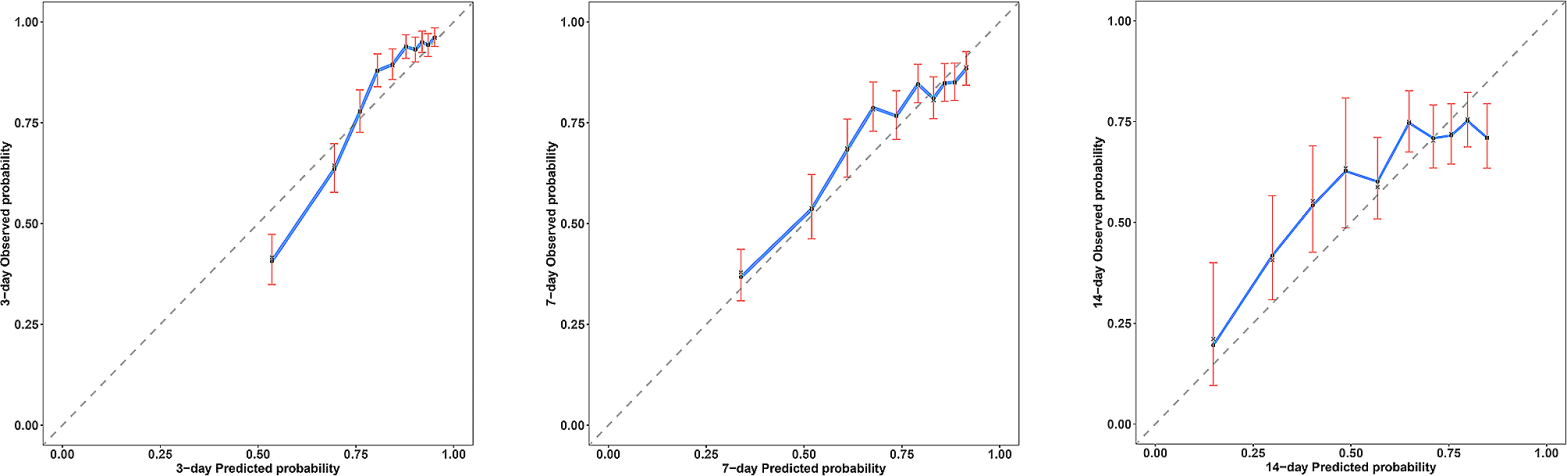
The calibration curve for the training set. (**A**) The calibration curve for the 3-day RF probability. (**B**) The calibration curve for the 7-day RF probability. (**C**) The calibration curve for the 14-day RP probability. RF, respiratory failure

**Fig. 5:**
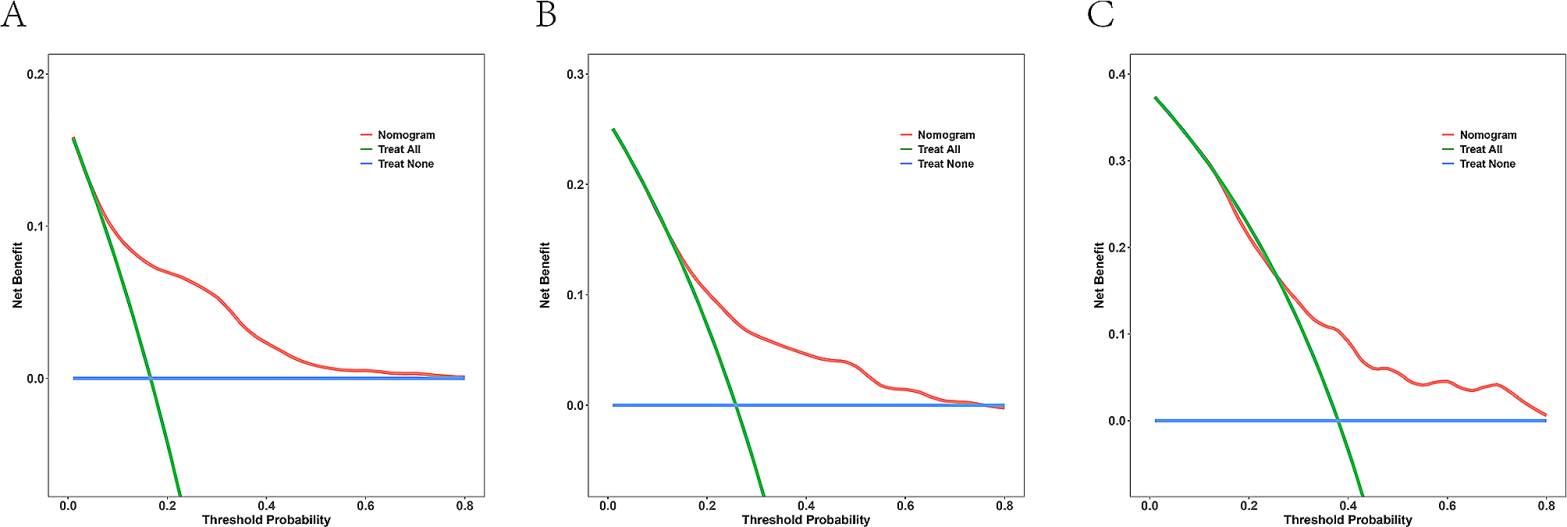
The DCA for the training set. (**A**) The DCA of 3-day. (**B**) The DCA of 7-day. (**C**) The DCA of 14-day. The blue (horizontal) line means that all samples are negative, and the green (oblique) line means that all samples are positive. The red line represents the risk nomograms. DCA, Decision curve analysis

**Fig. 6:**
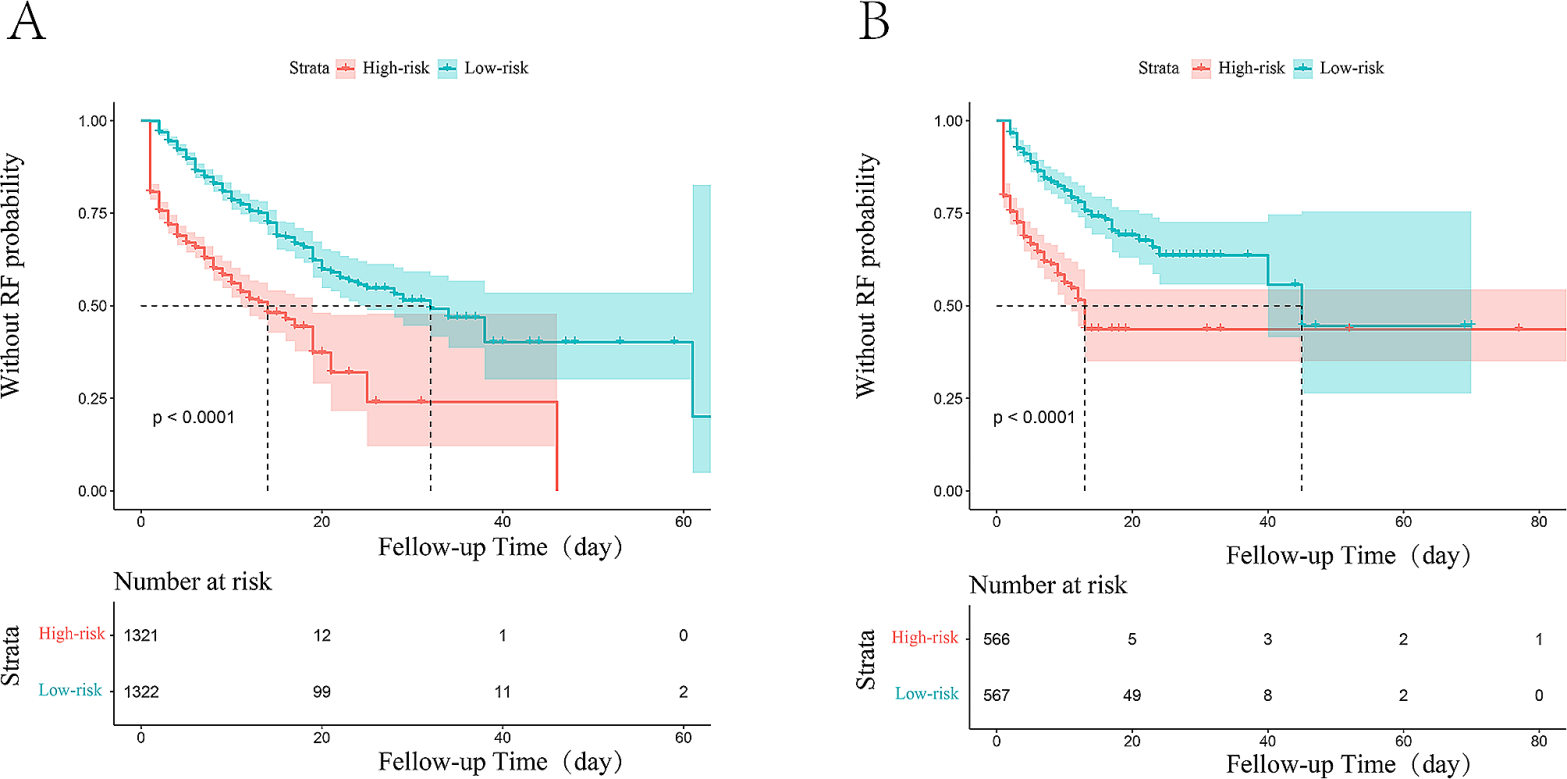
The K-M curves classified by low-risk group and high-risk group. (**A**) The K-M curve for the training set. (**B**)The K-M curve for the validation set. K-M curve, Kaplan-Meier curves

Calibration curves in the training set (Fig. 4A, B and C) and validation set (Figures S1A, S1B, S1C) were close to the diagonal, demonstrating good calibration of the model. The DCA curve (Training set: Fig. 5A, B and C, Validation set: Figures S2A, S2B, S2C) depicted the red line representing the scenario of predicting RF occurrence in AECOPD patients using the nomogram. For comparison, the green(oblique) and blue (horizontal) lines represent two extreme situations, with the green line indicating all positive samples and the blue line indicating all negative samples. The DCA curves demonstrated that in the training set, the model’s net benefit reached its maximum at threshold probabilities of 0.162 to 0.80 for 3 days, 0.258 to 0.751 for 7 days, and 0.382 to 0.825 for 14 days. In the validation set, the corresponding ranges were 0.174 to 0.562, greater than 0.26, and 0.177 to 0.664. The DCA results indicated that when predicting the probability of RF occurrence in AECOPD patients in the training set and validation set using the nomogram, we found that patients gained higher clinical net benefit. To assess the model’s reasonability, we calculated the total score for each patient and classified people with AECOPD into the low-risk and high-risk groups according to the median total score. Based on the K-M curve analysis (Fig. 6A and B), people with AECOPD in the high-risk group had a dramatically higher probability of RF occurrence than those in the low-risk group (*P* < 0.0001) in both the training and validation sets.

## Discussion

Nomograms, based on large-scale data analysis, are applied in intelligent patient management and can assist in the diagnosis and treatment of diseases. They present research results through intuitive graphics, representing a simple, reliable, and practical predictive tool [7]. Nomograms have been widely used in areas such as oncology and cardiovascular diseases, achieving favorable clinical application results [8, 9]. To the best of our knowledge, we have constructed the first predictive model with good predictive performance for the probability of RF occurrence in AECOPD patients. The AUC (95% CI) for the nomogram in the training set was 0.858 (0.836–0.881), 0.773 (0.746–0.799), and 0.736 (0.701–0.771) within 3, 7, and 14 days, respectively, and in the validation set, the AUC was 0.857 (0.822–0.892), 0.779 (0.739–0.819), and 0.777 (0.725–0.830), respectively. The predictive model established in this study demonstrated good discriminative ability (Fig. 3), calibration (Fig. 4), clinical impact (Fig. 5), and substantial discriminatory power (Fig. 6). Additionally, patient information in our study was extracted from MIMIC-IV 2.2, including 3,776 eligible patients, contributing to the generalizability of the results. Predictors such as BUN, PT, WBC, HR, comorbidities like ILD and RF, and the use of antibiotics and bronchodilators can be considered factors predicting RF occurrence in AECOPD patients. Therefore, by obtaining blood test indicators, vital signs, medication use, and comorbidities after patient admission, we can predict the probability of RF occurrence in AECOPD patients. This predictive model can guide the development of strategies to prevent RF in AECOPD patients.

BUN is an end product of protein metabolism, during which proteins in the body break down into amino acids, and after deamination of amino acids, ammonia is produced and detoxified in the liver, forming BUN [10]. Previous studies have shown that BUN, in addition to reflecting kidney function, also to some extent indicated muscle mass [11]. Our study revealed that BUN was an effective predictive indicator for the occurrence of RF in AECOPD patients. A higher BUN level had a higher nomogram score, indicating a higher probability of RF occurrence. This phenomenon may be attributed to the increased energy consumption and muscle protein breakdown in the acute phase of COPD, leading to an elevation in BUN. The increased energy consumption and muscle breakdown can cause intense inflammatory reactions and malnutrition, further resulting in skeletal muscle dysfunction [12]. Additionally, other studies suggest that muscle wasting can impact diaphragmatic fatigue [13], reduce respiratory muscle strength [14], and contribute to RF in AECOPD patients. The number of research on the correlation between decreased kidney function and RF is limited. A study reported that positive fluid balance was a risk factor for the inability of chronic critically ill patients to wean off mechanical ventilation [11]. Therefore, we speculate that declining kidney function may cause disturbances in fluid, electrolyte, and acid-base balance, leading to fluid accumulation in the lungs, causing pulmonary edema, and subsequently resulting in the occurrence of RF.

WBC is a marker indicating infection [15], and respiratory tract infections are common triggers for AECOPD and the occurrence of RF [16]. Infections may exacerbate COPD symptoms and lead to RF by causing airway inflammation, airway remodeling, increased airway reactivity, and bronchospasm [17]. Therefore, early control of infection may be a crucial measure to prevent RF in AECOPD patients. The 2017 ERS/ATS AECOPD guidelines [3] suggest that early use of antimicrobial agents can reduce treatment failure rates and delay AECOPD. For AECOPD outpatient patients, especially those with frequent exacerbations, the use of antimicrobial agents is recommended. Consistent with previous research, our study identified WBC and the non-use of antibiotics after admission as potential predictive factors for the occurrence of RF in AECOPD.

Our study indicated that concomitant HF and increased heart rate were potential risk factors for the development of RF in individuals with AECOPD. HF refers to a syndrome in which impaired cardiac pumping function due to various reasons leads to an insufficient cardiac output to meet the basic metabolic needs of the body’s tissues [18]. After the occurrence of HR, the following pathophysiological changes may occur [19, 20]: (1) weakened cardiac pumping function, reduced blood flow perfusion to different parts of the body, including the lungs; (2) obstruction of venous return in HF patients, leading to edema; (3) activation of the neuroendocrine system in the body, releasing a series of hormones. We speculate that the first two changes can lead to the occurrence of RF by affecting gas exchange in the lungs. After activation of the neuroendocrine system, hormones such as catecholamines are produced [20], excessive catecholamines can cause vasoconstriction mediated by α-1 adrenergic receptors, reducing blood flow to the alveoli, and can also cause vasodilation mediated by β2-adrenergic receptors, causing dilation of blood vessels around poorly ventilated alveoli. Additionally, catecholamines can increase HR, and an increased HR shortens ventricular filling time, reduces stroke volume, and decreases blood volume entering the lungs, leading to a mismatch in ventilation-perfusion ratio and increased intrapulmonary shunting, exacerbating the patient’s hypoxic state [21], and thus leading to the occurrence of RF.

Bronchodilators are among the main measures to control symptoms in AECOPD [22]. The use of bronchodilators can improve respiratory symptoms, reduce the risk of deterioration, increase exercise tolerance, and improve quality of life in patients [23]. Our study indicated that the non-use of bronchodilators after admission was a risk factor for the occurrence of RF in AECOPD patients. This suggests that early use of bronchodilators may lower the risk of deterioration in people with AECOPD.

ILD is a collection of diseases and has the characteristics of inflammation and fibrosis infiltration of the interstitium, which significantly alter the alveolar epithelium and capillary endothelium [24]. A study has pointed out that patients with ILD have a higher mortality rate compared with those with isolated COPD [25]. Our investigation is consistent with previous research, revealing that the presence of ILD is a risk factor for the occurrence of RF in people with AECOPD. We speculate that the underlying reason may be that the high expression of Klebs von den Lungen-6 (KL-6) in type II alveolar epithelial cells as well as bronchial epithelial cells of ILD patients, and KL-6 is associated with the severity as well as the progression of the disease. Additionally, IL-6 can promote the migration and proliferation of pulmonary fibroblasts [26, 27], causing restrictive ventilation dysfunction and consequently leading to the occurrence of RF.

Blood in AECOPD patients was found in a hypercoagulable state, and continuous progression can lead to pulmonary thrombosis. Early anticoagulation can significantly improve patients’ lung function, slow down disease progression, and reduce hospitalization time and frequency [28]. Therefore, preventive anticoagulation therapy may be adopted for COPD patients. PT is an indicator reflecting coagulation function, and anticoagulation treatment can lead to prolongation of PT. We found that prolonged PT was a risk factor for RF in AECOPD patients. This reminds us that the anticoagulation indications in patients should be assessed strictly, as anticoagulation treatment may contribute to disease progression and the occurrence of RF in AECOPD patients.

### Limitations

However, some limitations in the current research need to be addressed. Firstly, due to restrictions in the databases, our research did not include information on lifestyle and other related factors. Secondly, this is a single-center retrospective study, and statistical issues such as selection bias and uncontrolled confounding factors exist. Therefore, data from other sources are required for further validation of our findings. In conclusion, this study suggests that the nomogram may be effective for predicting the probability of RF occurrence in AECOPD patients. However, further prospective and multicenter studies are required to validate our findings.

## Conclusion

With 8 easily accessible parameters, the research has constructed and validated a nomogram for predicting the occurrence of RF in individuals with AECOPD after hospital admission. This nomogram can predict the probability of RF in AECOPD patients in the early stages after admission, offering valuable information for clinicians to take timely interventions, thereby improving patient prognosis.

### Electronic supplementary material

Below is the link to the electronic supplementary material.

Supplementary Material 1

## Data Availability

The data that support the findings of this study are available from the corresponding author upon reasonable request.

## Abbreviations

AECOPD: Acute exacerbation of chronic obstructive pulmonary disease
RF: Respiratory failure
MIMIC-IV: Medical Information Mart for Intensive Care IV
LASSO: Least absolute shrinkage and selection operator
ROC: Receiver operating characteristic
DCA: Decision-curve analysis
C-index: Concordance index
K-M: Kaplan-Meier
AUC: Areas under the curve
BIDMC: Beth Israel Deaconess Medical Center
ICU: Intensive Care Unit
SD: Standard deviation
IQR: Interquartile range
MSE: Mean square error
