# Supplementary material for: Development and validation of a model for predicting the early occurrence of RF in ICU-admitted AECOPD patients: a retrospective analysis based on the MIMIC-IV database
Source: BMC Pulm Med. 2024 Jun 26;24:302. doi: 10.1186/s12890-024-03099-2 (PMC11200819; doi:10.1186/s12890-024-03099-2)
Supplement: Supplementary file 1 — Supplementary Material 1 [file 12890_2024_3099_MOESM1_ESM.docx]

**Supplementary information**


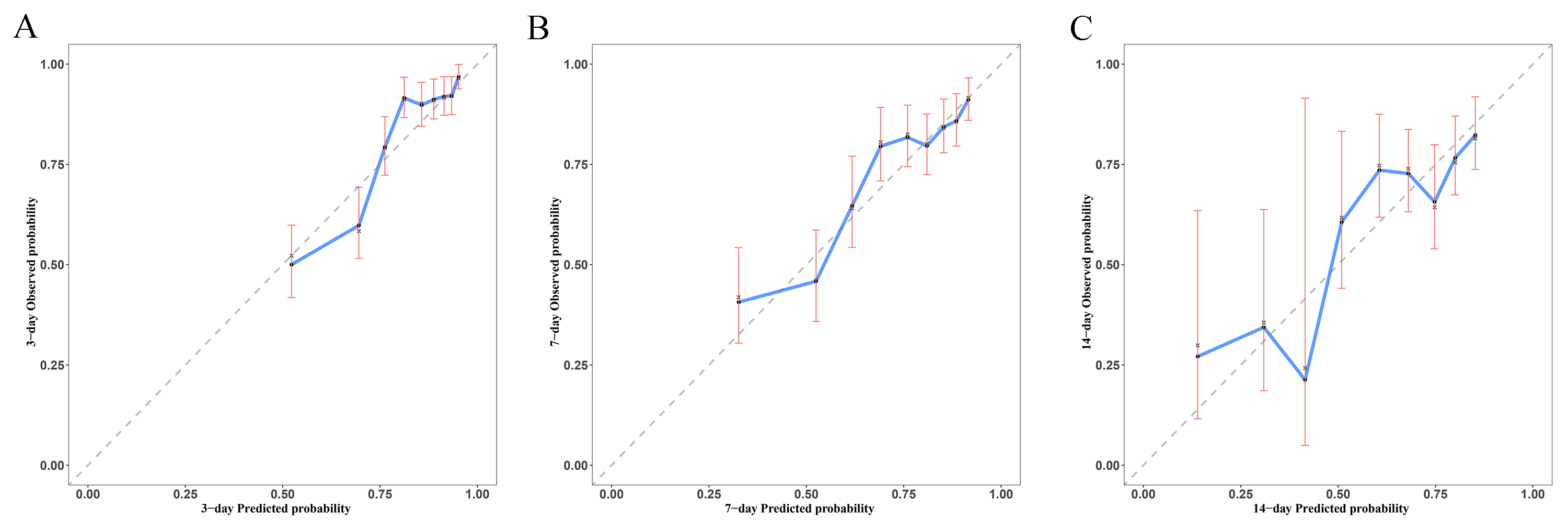


FIGURE S1

The calibration curve for the validation set. (A) The calibration curve for the 3-day RF probability. (B) The calibration curve for the 7-day RF probability. (C) The calibration curve for the 14-day RP probability. RF, respiratory failure


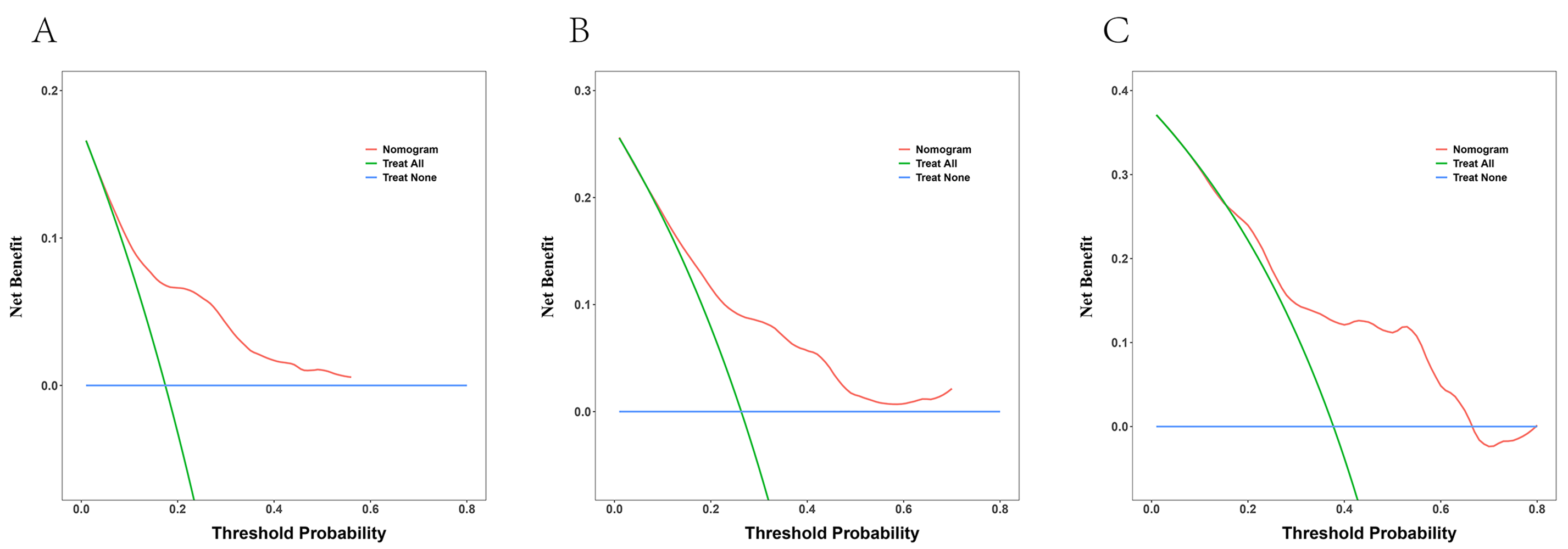


FIGURE S2

The DCA for the validation set. (A) The DCA of 3-day. (B) The DCA of 7-day.(C) The DCA of 14-day The blue (horizontal) line means that all samples are negative, and the green (oblique) line means that all samples are positive. The red line represents the risk nomograms. DCA, Decision curve analysis.

**Table S1 Results of normality test**

| Variables | P-value |
| --- | --- |
| Age | <2.2e-16 |
| APTT | <2.2e-16 |
| BUN | <2.2e-16 |
| Glucose | <2.2e-16 |
| HbA1c | 0.00628 |
| K+ | <2.2e-16 |
| Na+ | <2.2e-16 |
| PLT | <2.2e-16 |
| PT | <2.2e-16 |
| RBC | <6.861e-05 |
| Scr | <2.2e-16 |
| WBC | <2.2e-16 |
| HR | <2.2e-16 |
| RR | <2.2e-16 |

*Abbreviations*

HR, Heart rate; RR, Respiratory rate; WBC, White blood cell; RBC, Red blood cell; PLT; Platelet; HbA1c, Glycosylated hemoglobin; Scr, Serum creatinine; K+, Potassium; Na+, Sodium; BUN, Blood urea nitrogen; APTT, Activated partial thromboplastin time; PT, Prothrombin time

**Table S2:** **Missing proportion of each index**

| Serial number | Factors | Missing ratio |
| --- | --- | --- |
| 1 | Age | 0 |
| 2 | Gender | 0 |
| 3 | BMI | 0.512447 |
| 4 | Albumin | 0.50821 |
| 5 | ALT | 0.393273 |
| 6 | APTT | 0.106462 |
| 7 | AST | 0.390625 |
| 8 | BUN | 0.009799 |
| 10 | D-Dimer | 0.986494 |
| 11 | Glucose | 0.010593 |
| 12 | HbA1c | 0.010858 |
| 13 | HDL | 0.899894 |
| 14 | IBIL | 0.941737 |
| 15 | K | 0.008739 |
| 16 | Lcell | 0.796345 |
| 17 | LDL | 0.903072 |
| 18 | Na | 0.00821 |
| 19 | Ncell | 0.796345 |
| 20 | PLT | 0.012182 |
| 21 | PT | 0.097987 |
| 22 | RBC | 0.011123 |
| 23 | Scr | 0.009799 |
| 24 | TBil | 0.400424 |
| 25 | TC | 0.897246 |
| 26 | Triglycerides | 0.856992 |
| 27 | WBC | 0.012182 |
| 28 | Asthma | 0 |
| 29 | CVD | 0 |
| 30 | Diabetes | 0 |
| 31 | HF | 0 |
| 32 | ILD | 0 |
| 33 | Liver cirrhosis | 0 |
| 34 | Viral hepatitis | 0 |
| 35 | Bronchodilators | 0 |
| 36 | Usealbumin | 0 |
| 37 | GC | 0 |
| 38 | Antibiotic | 0 |
| 39 | HR | 0 |
| 40 | RR | 0.001589 |

*Abbreviations*

HR, Heart rate; RR, Respiratory rate; BMI, Body Mass Index; CVD, Cerebrovascular disease; HF, Heart failure; DM, Diabetes mellitus; WBC, White blood cell; Ncell, Neutrophile granulocyte; RBC, Red blood cell; PLT; Platelet; HbA1c, Glycosylated hemoglobin; Tbil, Total bilirubin; Ibil, Indirect bilirubin; ALT, Glutamic-pyruvic transaminase; AST, Glutamic oxalacetic transaminase; Scr, Serum creatinine; K^+^, Potassium; Na+, Sodium; BUN, Blood urea nitrogen; TC, Total cholesterol; LDL, Low Density Lipoprotein; HDL, High density lipoprotein; APTT, Activated partial thromboplastin time; PT, Prothrombin time; RF, Respiratory failure.
